# Berberine for Adjunct/Alternative Treatment of Dyslipidemia: A Literature Review

**DOI:** 10.7759/cureus.39261

**Published:** 2023-05-20

**Authors:** Endurance O Evbayekha, Elochukwu U Nwachukwu, Elham Nikravesh, Valene Rosas, Chinwendu A Onuegbu, Obinna F Egwuonwu, Osazee Eguagie, Ogochukwu E Chioma, Awanwosa V Agho, Kemar A Samuels, Anthony Willie, Jane N Nwafor, Laura N Esene-Akhideno, Aisha O Adigun

**Affiliations:** 1 Internal Medicine, St. Luke's Hospital, Chesterfield, USA; 2 Family Medicine, University of Uyo Teaching Hospital, Uyo, NGA; 3 Family Medicine, Guilan University of Medical Sciences, Rasht, IRN; 4 Psychiatry, MCR Behavioral Health Services, Temecula, USA; 5 Internal Medicine, Montefiore Medical Center, Bronx, USA; 6 Family Medicine, University of Nigeria Teaching Hospital, Enugu, NGA; 7 Medicine, St. George's University, True Blue, GRD; 8 Surgery, Federal Medical Center Keffi, Keffi, NGA; 9 Internal Medicine, University of Benin, Benin, NGA; 10 Internal Medicine, Escuela Latinoamericana de Medicina, Havana, CUB; 11 Emergency Medicine, Igbinedion University Okada, Benin, NGA; 12 Internal Medicine, University of the District of Columbia, Silver Spring, USA; 13 School of Medicine, American University of St. Vincent, Kingstown, VCT; 14 Infectious Diseases, University of Louisville, Louisville, USA

**Keywords:** inflammation, atherosclerosis, dyslipidemia, berberis spp, berberine, berberidaceae

## Abstract

Berberine (BBR) is an ancient plant popular in China and is used to treat dyslipidemia, among other cardiovascular and metabolic-related diseases. BBR has historically been regarded as having multiple benefits, with a few clinical trials indicating this fact. We searched PubMed, Embase, and Google Scholar with the following keywords: Berberidaceae, berberine, Berberis spp., dyslipidemia, atherosclerosis, and inflammation. We synthesized the information within the literature to provide an updated review of BBR, its potential, and its applicability in real-world medicine in the future. This review sought to evaluate the literature and advancement in BBR’s efficacy regarding dyslipidemia, inflammation, and atherosclerosis.

## Introduction and background

Statins (STs) are the current standard of treatment for dyslipidemia and atherosclerosis. The question about whether they are the only option with that efficacy has been frequently asked. The proprotein convertase subtilisin/kexin type 9 (PSK9) inhibitors have recently become an established alternative or add-on medication in some subgroups of patients. Recently, bempedoic acid gained some popularity due to early analysis results revealing that it may suit individuals with ST intolerance [1]. Issues regarding the affordability of the alternatives to STs have also been raised. Berberine (BBR) is a plant alkaloid used in traditional medicine in some countries, notably China [2]. Dyslipidemia is a highly prevalent and often asymptomatic condition, and it correlates with the high burden of cardiovascular morbidity and mortality in the United States and globally [1].

The treatment of choice for dyslipidemia is with STs in modern medicine. STs have shown great promise and are very efficacious. They also decrease morbidity and mortality [1], making them the preferred choice for patients with dyslipidemia and some cardiovascular conditions [3]. Like all medications, STs are far from perfection, with some problems associated with STs, such as GI intolerance and muscle manifestations, including muscle pain, fatigue, and weakness, elevated creatine phosphokinase (CPK), and in some cases, increased liver transaminase. However, the prevalence of ST intolerance is low, reportedly ranging from 7% to 30% [4]. BBR is of the genus Berberis (Berberidaceae). It consists of 594 species that have had remarkable results when used for various pathological conditions [5]. For the sake of this literature, we focused on its benefit in patients with dyslipidemia and atherosclerosis. Several studies have highlighted the beneficial effects of BBR. This is reportedly due to the isoquinoline alkaloid, which it contains [5,6].

The role of inflammation in atherogenesis has been clearly stated in various pieces of literature. Inflammatory damage to the endothelial lining of vascular structures triggers a cascade of events leading to the release of interleukin (IL) 1, IL-6, tumor necrosis factor-alpha (TNF-α), the accumulation of lipid-laden macrophages, and a vicious cycle that ultimately causes the thinning of the plaque capsule leading to a coronary event or a thromboembolic phenomenon. BBR has been shown to have antiobesity, cardioprotective, anti-inflammatory, anti-atherosclerotic, anti-lipidemic, and anti-diabetic properties [6-10].

The mechanism through which BBR acts is poorly understood. However, BBR has been used in various traditional medicines with demonstrated benefits for multiple diseases, especially cardiovascular health. Various clinical trials have demonstrated its efficacy in treating hyperlipidemia in individuals with or without comorbidities such as coronary heart disease and diabetes mellitus [8,9]. Because of the spectrum of cardiovascular-related pathologies that BBR could benefit from, it is crucial to explore the possibilities with an open mind. Our study aimed to review the underexplored yet beneficial effects of BBR on dyslipidemia, inflammation, and atherosclerosis and highlight gaps and clinical scenarios where BBR’s efficacy can be harnessed to improve the evidence-based management approach for these pathologies.

## Review

Methodology

We searched PubMed, Google Scholar, ScienceDirect, and Embase based on the defined inclusion and exclusion criteria. We included articles from the last 22 years (2000 to 2022). Keywords for the search included Berberidaceae, berberine, Berberis spp., dyslipidemia, atherosclerosis, and inflammation. Using the search engine layout, we made keyword combinations to generate the included articles and made every possible combination of the search keywords.

**Figure 1:**
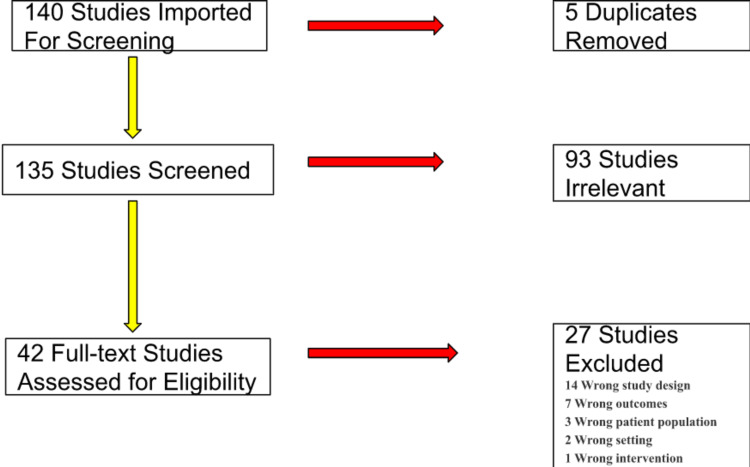
Illustration of the literature search process

Our keyword inquiry resulted in 140 articles from various repositories. Initially, we screened the results by reviewing their titles, yielding 121 matches. Subsequently, we read abstracts and narrowed the articles to 42 studies in full text, represented in Figure 1 below.

Eligibility criteria

We reviewed various original full-text articles, randomized clinical trials, and cohort studies focused on BBR’s mechanism of action, anti-inflammatory actions within the vascular system, and lipid-lowering effects. We defined inflammation, dyslipidemia, and atherosclerosis using the WHO definition [1]. We excluded non-English translated articles, those without an available full text, those unrelated to BBR, dyslipidemia, inflammation, and atherosclerosis, and those outside the specified year range. We summarized our methodological process in Figure 1.

Mechanism of action of BBR

BBR’s potency is derived from the alkaloid within. Some literature has suggested its mechanism of action is via the attenuation of nuclear factor kappa B (NF-κB) translocation. One study demonstrated BBR’s effect on arachidonic acid (AA) and lipopolysaccharide (LPS)-induced activation on inflammatory markers such as TNF-α, monocyte chemoattractant-1 (MCP-1), IL-8, IL-6, cyclooxygenase-2 (COX-2), and NF-κB translocation into the nucleus of the T-helper cell-1 (THC-1) [11]. It revealed a marked reduction in the potency of inflammatory mediators and inhibition of NF-κB translocation, consequently serving as an effective anti-inflammatory agent against various chronic inflammatory conditions, including atherosclerosis. The mechanism through which BBR acts to correct dyslipidemia works through the degradation and ubiquitination of hepatocyte nuclear factor 1 alpha (HNF-1α), resulting in an inhibitor of PCSK9. This causes a reduction in the lysosomal degradation of hepatic low-density lipoprotein receptors (LDLR) [10].

Furthermore, BBR directly affects the expression of LDLR, leading to an upregulation of the receptors via a mechanism that stabilizes LDLR messenger ribonucleic acid (mRNA) [12]. Recent studies indicate that BBR attenuates intestinal cholesterol absorption, increasing fecal excretion and hepatic cholesterol turnover. Xiaoyan Qiang et al. concluded that BBR enhanced cholesterol excretion from the liver to the bile in hamsters with hyperlipidemia, resulting in a significant decrease in the serum total cholesterol (TC), triglyceride (TG), and low-density lipoprotein cholesterol (LDL-C) levels [13].

BBR’s effects on the serum transaminases were negligible, reflecting a significant tendency to cause liver dysfunction [14-16]. The curiosity behind BBR stems from its potential to go beyond treating dyslipidemia or atherosclerosis. It shows great promise for chronic inflammatory diseases, which cover the most prevalent diseases in today’s America, such as obesity [17,18]. Although the mechanism is through a different pathway, BBR was studied to alleviate fatty liver in obese animal subjects by activating adenosine monophosphate-activated protein kinase (AMPK) in peripheral tissues culminating in an increase in fatty acid oxidation [19,20]. AMPK is crucial in energy balance and metabolism at the cellular level. Activating AMPK is a key innate response to inflammation [16,21]. This study indicates that BBR has the potential for effective therapy in diabetes mellitus, dyslipidemia, and inflammatory pathologies, especially atherosclerosis, with no reported significant side effects. The low cost of BBR, compared with other first-line medications, puts BBR in a great position to be an alternative for patients with these conditions [22,23].

Dyslipidemia

Dyslipidemia is referred to the alteration of plasma lipid levels, particularly an abnormal increase in total cholesterol and/or triglycerides, apolipoprotein-a, or low-density lipoprotein (LDL). Dyslipidemias are among the most common and easily detected and treated chronic conditions.

An associated clinical consequence of dyslipidemia is an increased risk for atherosclerotic cardiovascular disease (ASCVD). Additional predisposing factors, especially obesity and/or type 2 diabetes, are present in most cases. Recent research studies have provided insight into the biomolecular and genetic basis of dyslipidemias, highlighting their role in the development of atherosclerosis. Pharmacological management options are also expanding, including the use of monoclonal antibodies that target PCSK9. A recent clinical trial reports bempedoic acid in ST-intolerant individuals [24]. Derosa et al. conducted a study on young women and men between the ages of 21 and 27 years. These individuals received a daily BBR dose of 1176 mg associated with silymarin for 90 days. BBR effectively reduced total cholesterol in this study to −4.84 (−5.39 to −4.28) [9]. A similar study was conducted by Orio et al. but with an older population with a median age of 50-60 years, which supports Derosa et al.’s findings [9,23].

In another randomized clinical study where there were 692 participants in the control group and 733 in the treatment group, after adjusting for confounders, the total cholesterol in the treatment group had decreased significantly by 1.06 (0.64, 1.48) compared to the placebo group (p = 0.00) [25].

In a randomized study that consisted of 660 participants in the placebo group and 701 in the treatment arm, there was a significant decrease in the LDL concentration in the treatment group (1.77 (1.11, 2.44); p = 0.00) when compared to the control group [25]. Although the bioavailability of oral BBR is low, the oral dosage needed to cause a significant change in serum lipid is relatively small. This is a pointer that the GI requires only a few doses to cause significant effects; this suggests that the intestinal tract is a potentially effective site of action for the hypolipidemic effects of BBR [13,15]. With purification and tablet formation, future drug production could decrease the dosage needed to cause significant lipid-lowering effects and lower the incidence of drug side effects.

Jiarong Lan et al. conducted a meta-analysis of 27 clinical studies analyzing the lipid-lowering potency of BBR for LDL-C, high-density lipoprotein cholesterol (HDL-C), and TG. This resulted in a clinically significant finding [23]. This was similarly supported by a preclinical study conducted on laboratory rats. BBR reduced serum cholesterol levels in diet-induced hypercholesterolemic rats. This was partly due to intestinal cholesterol absorption inhibition due to BBR's ability to inhibit intraluminal cholesterol micellization and enterocyte cholesterol uptake [7]. To summarize the highlights, Li et al. concluded that BBR improves intestinal health, systemic inflammation, and atherosclerosis in various ways, as shown in Table 1 below [16].

**Table 1:** Berberine's impact on intestinal health and systemic inflammation

Reduces intestinal permeability	Increases intestinal tight junction protein expression, colonic mucinous layer thickness, levels of *Akkermansia*, and short-chain fatty acids
Reduces plasma lipopolysaccharide (endotoxemia)	Decreases pro-inflammatory cytokines and systemic inflammation
Improves liver insulin resistance	Decreases liver flavin monooxygenase 3 (FMO3) expression

Inflammation

Inflammation is the hallmark of various diseases in our society today. The etiology of various pathologies could be traced back to inflammation playing a key role in their pathogenesis. BBR’s ability to fight against inflammation and its hypolipidemic actions are among the strong points for combating atherosclerosis. The two-prong attack makes BBR desirable for clinical medicine and worthy of further studies to explore its outstanding potential. BBR’s treatment inhibited inflammation in cells by triggering autophagy, which was carried out by activating the AMPK/mammalian target of rapamycin (mTOR) signaling pathway [22]. One of the mechanisms through which intracellular homeostasis is maintained is autophagy. Autophagy is the process in which damaged organelles are removed during various cell stresses [21]. BBR also encourages healthy intracellular balance while acting as a protective agent against various diseases.

Jiang et al. revealed that BBR could be used to treat inflammation and cancer, and its therapeutic uses have been growing in popularity. BBR has a molecular weight of 336.337 g/mol and the chemical formula C20H18NO4. It is primarily metabolized in the liver and gut [26]. BBR inhibits the synthesis of cellular reactive oxygen species and mitogen-activated protein kinase signaling to decrease proinflammatory responses [27]. Recent studies have shown that BBR exerts anti-inflammatory effects in the intestinal lumen by controlling the transcription of these substrates. This ameliorates intestinal epithelial damage brought on by pro-inflammatory cytokines, primarily achieved by activating AMPK and inhibiting transcription factor activator protein 1 (AP1) and NF-κB [28]. BBR is a pharmaceutical plant derivate that can be grown globally [29]. BBR can also cause DNA damage, telomerase suppression, topoisomerase poisoning, modify mitochondrial membrane, control Bcl-2 family members, and block cell signaling pathways [30].

BBR in hyperuricemia

Hyperuricemia is a pathologic state when blood uric acid levels are abnormally high. The normal serum upper limit in men is 7 mg/dl, and in women is 6 mg/dl. An elevated level of uric acid is a driving factor for the development of gout. It has been linked to multiple renal pathologies that may lead to chronic nephropathy, an important state of chronic inflammation, and accelerated heart disease. In a mice animal model study, using step-wise increasing doses of BBR lowered serum uric acid levels with significant results (p < 0.001) in all three dosages. This was due to BBR’s ability to induce increased urinary uric acid levels and fractional urate excretion [31].

BBR in polycystic ovarian syndrome

Polycystic ovary syndrome (PCOS) is a constellation of endocrinopathy characterized by infertility, menstrual dysregulation, hyperandrogenemia, acanthosis nigricans, and a hormone profile revealing increased luteinizing hormone/follicle-stimulating hormone (LH/FSH) ratio, hyperinsulinemia, elevated androgen levels, obesity, and dyslipidemia. Mishra et al. studied the effects of BBR, metformin, and myoinositol in women with PCOS. Subjects were randomized into three groups. One group received BBR hydrochloride 500 mg twice daily, the second received metformin hydrochloride 500 mg twice daily, and the third group got myoinositol 1000 mg twice daily [32].

They evaluated the mean weight, waist-to-hip ratio, waist circumference, and body mass index. They also evaluated metabolic parameters such as serum fasting insulin, fasting blood sugar, fasting blood sugar to serum fasting insulin (FBS/FI) ratio, sex hormonal effects, and impact on lipid profile. There was an improvement in all parameters between the three groups. However, BBR showed a greater difference in hormonal, clinical, and lipid parameters than myoinositol and metformin. They concluded that BBR might have greater potential to decrease cardiovascular risk than metformin in PCOS patients. This was due to BBR’s positive effect on lipid profile, body composition, and hormone status [32].

Side effects of BBR

Interestingly, some documented side effects from BBR clinical trials were primarily mild to moderate, predominantly gastrointestinal (diarrhea and constipation), and relatively similar in frequency to the control groups. Significant side effects, such as creatinine elevation or liver enzyme levels relative to the control group, were not reported [9,17,24].

Limitations

Despite the abundance of preclinical studies supporting its efficacy in various diseases, there is a paucity of clinical studies on human subjects. The heterogeneity of the studies, sample size, and scarcity of follow-up data are limiting factors. The overall limited quality of the included studies limits the precise interpretation of the beneficial effects of BBR to some degree. Improved methodological quality and large controlled trials using standardized preparation will mitigate this problem.

## Conclusions

BBR is an isoquinoline alkaloid with a multifaceted mechanism of action that reflects its broad-spectrum potential and application profile. With the demonstrated potency for GI absorption, pharmaceutical strategies to improve GI absorption and aqueous solubility will likely unlock its pharmacological potential. Clinical studies in humans are, however, few and far between. Hence, more prospective studies emphasizing the Framingham score change and C-reactive protein with patient follow-up are required to provide more detailed information on BBR’s efficacy in treating dyslipidemia, atherosclerosis, and overall cardiovascular benefit in the long run. Furthermore, to calculate BBR's safety profile, more clinical studies on human subjects should be encouraged to evaluate and detect the rare adverse effects of BBR and to strengthen its applicability.
